# Upregulated gga-miR-16-5p Inhibits the Proliferation Cycle and Promotes the Apoptosis of *MG*-Infected DF-1 Cells by Repressing PIK3R1-Mediated the PI3K/Akt/NF-κB Pathway to Exert Anti-Inflammatory Effect

**DOI:** 10.3390/ijms20051036

**Published:** 2019-02-27

**Authors:** Kang Zhang, Yun Han, Yabo Zhao, Yingfei Sun, Mengyun Zou, Yali Fu, Xiuli Peng

**Affiliations:** Key Laboratory of Agricultural Animal Genetics, Breeding and Reproduction, Ministry of Education, College of Animal Science and Technology and College of Veterinary Medicine, Huazhong Agricultural University, Wuhan 430070, China; zhangkang123@webmail.hzau.edu.cn (K.Z.); Hany@webmail.hzau.edu.cn (Y.H.); zyb@webmail.hzau.edu.cn (Y.Z.); sunyingfei@webmail.hzau.edu.cn (Y.S.); zoumengyun@webmail.hzau.edu.cn (M.Z.); FYL@webmail.hzau.edu.cn (Y.F.)

**Keywords:** *MG*, chicken, PI3K/Akt/NF-κB pathway, gga-miR-16-5p

## Abstract

*Mycoplasma gallisepticum* (*MG*) mainly infects chickens to initiate chronic respiratory disease (CRD). microRNAs (miRNAs) play vital roles according to previously reported studies. Our previous study showed that gga-miR-16-5p, in *MG*-infected lungs of chicken embryo, was upregulated by Illumina sequencing. The study aimed to reveal what role gga-miR-16-5p plays in CRD progression. gga-miR-16-5p was upregulated in *MG*-infected fibroblast cells (DF-1). Phosphoinositide-3-kinase regulatory subunit 1 (PIK3R1) was demonstrated as the target gene of gga-miR-16-5p. Furthermore, PIK3R1 expression was lower in *MG*-infected groups than it in noninfected controls measured by qPCR. Additionally, overexpressed gga-miR-16-5p could downregulate PIK3R1 and phosphorylated serine/threonine kinase (p-Akt) to express protein, whereas there is an opposite effect on inhibition. Overexpressed gga-miR-16-5p resulted in decreased activity of tumor necrosis factor alpha (TNF-α) and the nuclear factor-kappaB (NF-κB) by qPCR. Furthermore, overexpressed gga-miR-16-5p restricted cell multiplication, cycle progression, and increased apoptosis of *MG*-infected DF-1 cells, whereas inhibited gga-miR-16-5p led to the opposite effect. Collectively, upregulated gga-miR-16-5p could decrease multiplication, cycle progression, and increase apoptosis of *MG*-infected DF-1 cells, at least partly through directly targeting PIK3R1 and inhibiting PI3K/Akt/NF-κB pathway to exert an anti-inflammatory effect. Our results will provide more experimental evidence to bring pathogenesis of *MG* infection to light.

## 1. Introduction

Mycoplasma is the smallest and simplest prokaryote with a large number of species in poultry, such as *Mycoplasma meleagridis*, *Mycoplasma iowae*, *Mycoplasma synvoviae* and *Mycoplasma gallisepticum* (*MG*) in poultry. *MG* infection mainly causes chronic respiratory disease (CRD) in chickens, by colonizing the respiratory mucosa and causes a severe inflammatory reaction and an immune response [1,2]. It is shown that *MG* is resistant to antibiotics and can evade the immune system, and eventually leads to systemic infection through the respiratory mucosal barrier [3]. CRD has become a major infectious disease, causing huge losses to the chicken industry [1]. The virulent strains used in this study were *MG-HS* strains isolated from henneries in Hubei, China [4,5].

Mature miRNAs are formed by a stem ring structure of miRNAs precursor processed by nuclease Dicer. As a cluster of endogenous single-stranded noncoding RNA; the miRNAs are only 22–25 nucleotides in length. miRNAs are ubiquitous in eukaryotes, and relatively conservative in species evolution, and highly homologous among different species. Mature miRNAs have several target genes, binding with their mRNA 3’ untranslated regions (3’ UTRs), causing translation inhibition and/or corresponding transcript degradation [6]. In recent years, accumulating evidence indicates that miRNAs are involved in multiple physiological and disease processes, consisting of proliferation, apoptosis, cycle progression of cells, and microbial infection [1,7]. It has been reported that the altered expression of miRNAs acts in critical roles in poultry diseases, for example, Marek’s disease [8,9,10,11], avian influenza [12], infection bursal disease [13], and avian leucosis [14,15]. Our current studies showed that some miRNAs are involved in CRD progression [16,17,18]. Overexpress of gga-miR-101-3p significantly inhibits EZH2 expression; EZH2 can positively regulate MAPK activity and cell proliferation [18]. gga-miR-19a suppress the expression of ZMYND11 and promotes NF-κB, MyD88, and TNF-α expression [17]. Upregulation of miR-130b-3p activates the PI3K/AKT/NF-κB pathway, facilitates cell proliferation and cell cycle via downregulating PTEN [19]. Interestingly, these results show that PI3K\p-Akt\NF-κB is an important pathway in MG infection. When we focused on this pathway, we found miR-16 may take part in the regulation of PIK3R1 expression [20].

The miR-16, a member of the miR-15a/16 gene cluster, is highly conserved and widely expressed. miR-16 was markedly downregulated in human nasopharyngeal carcinoma cells [21]. miR-16 had a significantly lower expression level in normal colorectal tissue than that in colorectal cancer patients [22]. miR-16 is not only related to the proliferation of cancer cells and viral replication, but also to many inflammatory reactions [23]. miR-16 can control the interaction between macrophages and the activity of T cells [24]. In many cancers, it has been recognized that miR-16 has a significant anticancer effect by affecting apoptosis, cycle, and proliferation of cells [25,26,27,28,29,30,31]. miR-16-5p also plays an anti-inflammatory role in lung inflammation caused by lipopolysaccharide [32]. However, little is known about the function and potential mechanism of gga-miR-16-5p in *MG* infection. Our pilot study presented that gga-miR-16-5p expression was significantly upregulated in embryonic lungs infected by *MG* according to Solexa deep sequencing data [33]; therefore, we speculate that gga-miRr-16-5p may play a role in *MG*-HS infection. Our study further validated that gga-miR-16-5p was remarkably upregulated in DF-1 cell lines and lungs of chicken embryos infected by *MG*. PIK3R1 was demonstrated as a direct functional target gene to gga-miR-16-5p. In addition, upregulated gga-miR-16-5p inhibits cell multiplication, the cell cycle of DF-1 cell lines infected by *MG*, and promotes cell apoptosis by inhibiting the PI3K/Akt/NF-κB pathway. In conclusion, the results suggest that gga-miR-16-5p inactivation of the PI3K/Akt/NF-κB pathway was related to *MG* infection and might be a target for miRNA-based treatment for CRD for the further study.

## 2. Results

### 2.1. gga-miR-16-5p Expression Was Markedly Upregulated in Lungs of Chicken Embryonic and DF-1 Cell Lines with MG Infection

**Figure 1:**
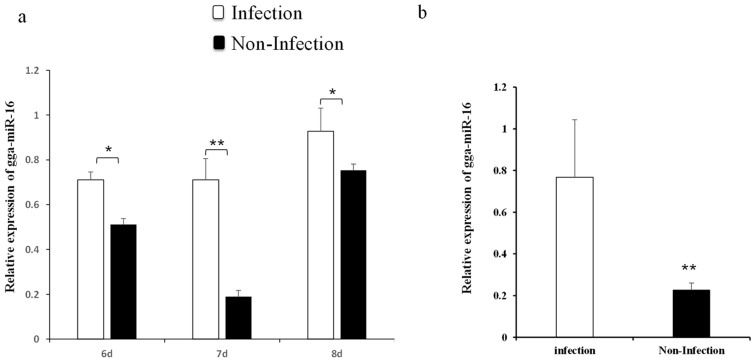
Expression of gga-miR-16-5p in DF-1 cells and chicken embryo lungs with and without *Mycoplasma gallisepticum* (*MG*) infection. Expression of gga-miR-16-5p was detected by RT-qPCR. Samples were normalized to 5S-rRNA. The clean lung tissues of chicken embryos and DF-1 cells were acted as negative controls. All data from the experiments carried out independently at three times were adopted as mean value ± SD and * *p* < 0.05, ** *p* < 0.01 indicated significant differences. The expression of miR-16-5p on the 6th–8th days postinfection in tissues (**a**) and DF-1 cells (**b**).

Our previous miRNAs deep sequencing data revealed gga-miR-16-5p was significantly upregulated in chicken embryonic lungs with *MG* infection [33]. To further confirm the result, the expression level of gga-miR-16-5p after *MG* infection was detected by qPCR. On the 6th, 7th, and 8th days postinfection (amount to the egg hatching 15th, 16th, and 17th days), the expression of gga-miR-16-5p was remarkably upregulated in *MG*-infected embryonic chicken lungs than that in noninfected lungs of chicken embryos (Figure 1a). Similar results were also shown in DF-1 cells (Figure 1b). Therefore, we can reasonably assume that gga-miR-16-5p directly participated in the pathogenesis of *MG* infection.

### 2.2. PIK3R1 Is a Direct Target Gene of gga-miR-16-5p in CRD of Chicken

**Figure 2:**
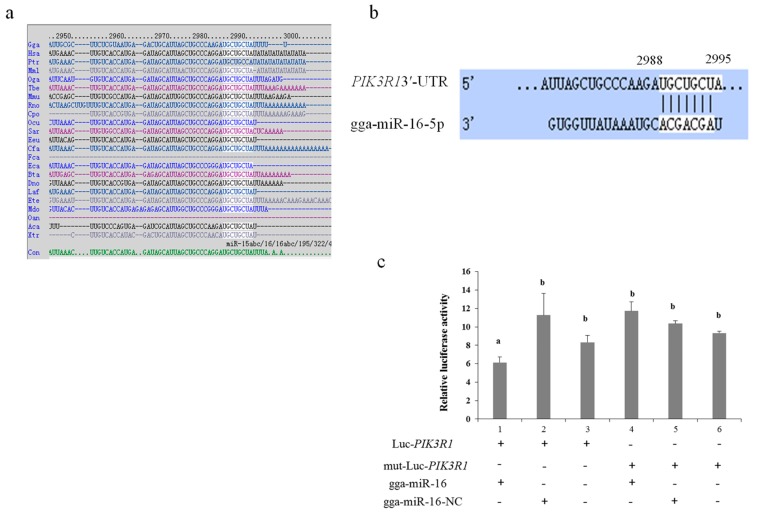
PIK3R1 is the direct target of gga-miR-16-5p. (**a**) Alignments of PIK3R1 3′-UTR derived from several species. The highlighted U to A sequence is the conserved target region. (**b**) Sequence alignments of gga-miR-16-5p. Position 2988–2995 in the 3′-UTR of PIK3R1, which is highlighted, was predicted to be the target site of it. The seed sequence in gga-miR-16-5p is also highlighted. (**c**) The recombinant plasmid and gga-miR-16-5p mimics were cotransfected into DF-1 cells. The cells were assayed firefly and Renilla luciferase by dual-luciferase assay transfected 24 h later. All data from the triplicate experiments carried out independently were adopted as mean value ± SD. (Different lowercase letters between groups mean *p* < 0.05.)

The function of miRNAs is to regulate their downstream target genes [34]. We found about 150 potential targets of gga-miR-16-5p using miRDB and TargetScan. Finally PIK3R1 was chosen because of its important roles in cell functions and inflammatory response. The target site sequence in the MAP3K1 3’-UTR was highly conserved in 2988–2995 bps among different species (Figure 2a,b).

To further validate that gga-miR-16-5p was able to combine with the PIK3R1 3’ UTR sequence directly, a luciferase reporter was constructed which contained the gga-miR-16-5p binding site or the corresponding mutant. According to the result of luciferase reporter assays (psi-CHECK™-2), the overexpression of gga-miR-16-5p decreased luciferase activity of PIK3R1 markedly, whereas there was no significant effect of gga-miR-16-5p transfection on luciferase activity of mut-luc-PIK3R1 (Figure 2c).

**Figure 3:**
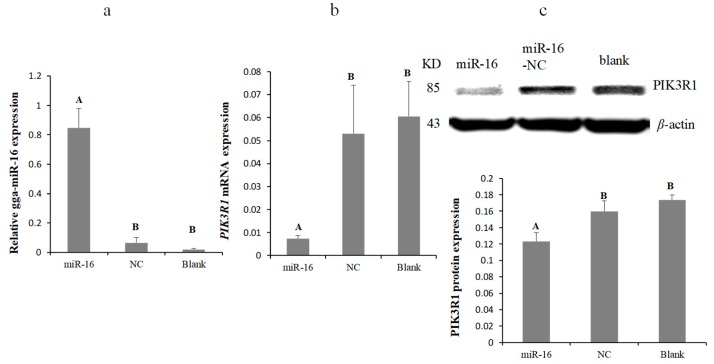
Overexpress of gga-miR-16-5p repressed PIK3R1 expression. (**a**) The detection results of gga-miR-16-5p overexpression. (**b**) Relative expression of PIK3R1 mRNA with gga-miR-16-5p overexpressed in DF-1 cells. GAPDH served as a loading control. (**c**) Relative expression levels of PIK3R1 protein in DF-1 cells with gga-miR-16-5p overexpressed. β-actin served as a loading control. All data from the triplicate experiments carried out independently were adopted as mean value ± SD. The significant difference (*p* < 0.01) is expressed with the capital letters and no difference with the same capital letters (*p* > 0.05).

**Figure 4:**
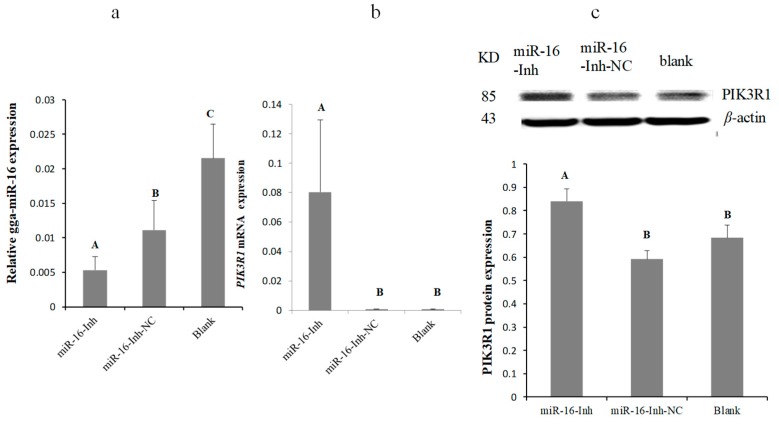
Inhibition of gga-miR-16-5p increased PIK3R1 expression. (**a**) Expression levels of gga-miR-16-5p with miR-16-Inh transfections. (**b**) Relative mRNA expression of PIK3R1 with miR-16-Inh transfections. GAPDH served as a loading control. (**c**) Relative protein expression of PIK3R1 post-transfected with miR-16-Inh at 48 h. β-actin served as a loading control. A mock-vehicle transfection was the blank group. All data from the triplicate experiments carried out independently were adopted as mean value ± SD. There was significant difference (*p* < 0.01) for different capital letters and no difference for the same capital letters (*p* > 0.05).

To further verify the mutual effect between the PIK3R1 3′-UTR and gga-miR-16-5p, miR-16 mimics, miR-16-NC, miR-16-Inh, or miR-16-Inh-NC were transfected into DF-1 cells, to validate the expression of endogenous PIK3R1 using Western blot and qPCR. gga-miR-16-5p was overexpressed by transient transfection (Figure 3a). PIK3R1 mRNA and protein expression levels both were significantly downregulated by the overexpression of gga-miR-16-5p after 48 h transfection (Figure 3b,c). On the contrary, the gga-miR-16-5p inhibitor inhibited the expression of gga-miR-16-5p noticeably (Figure 4a), leading to a significant increase in PIK3R1 expression on mRNA levels and protein levels (Figure 4b,c). These suggest that PIK3R1 is a direct target gene of gga-miR-16-5p which is negatively regulated by binding to PIK3R1 3′-UTR.

### 2.3. MG Infection Downregulates PIK3R1 Expression

**Figure 5:**
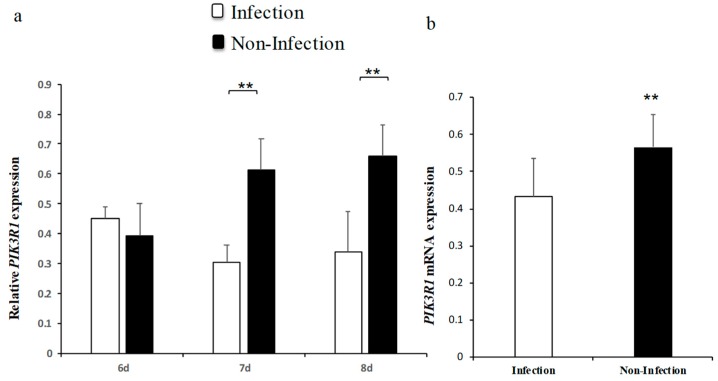
The relative expression of PIK3R1 in chicken embryo lung tissues and DF-1 cells. All samples were normalized to GAPDH. The uninfected DF-1 cells and lung tissues served as negative controls. The expression of PIK3R1 in the lung tissues on the 6th–8th days postinfection (**a**) and in DF-1 cells (**b**). All data from the triplicate experiments carried out independently were adopted as mean value ± SD. ** *p* < 0.01.

To further identify the expression pattern of PIK3R1 in *MG*-HS infection comprehensively, its expression levels in DF-1 cells and lung tissues were measured. It was found that PIK3R1 expression levels were significantly decreased on 7th and 8th days postinfection (Figure 5a,b). These results further demonstrate that PIK3R1 levels were inversely correlated with gga-miR-16-5p and PIK3R1 is repressed by gga-miR-16-5p.

### 2.4. gga-miR-16-5p Suppresses the PI3K/Akt Pathway

**Figure 6:**
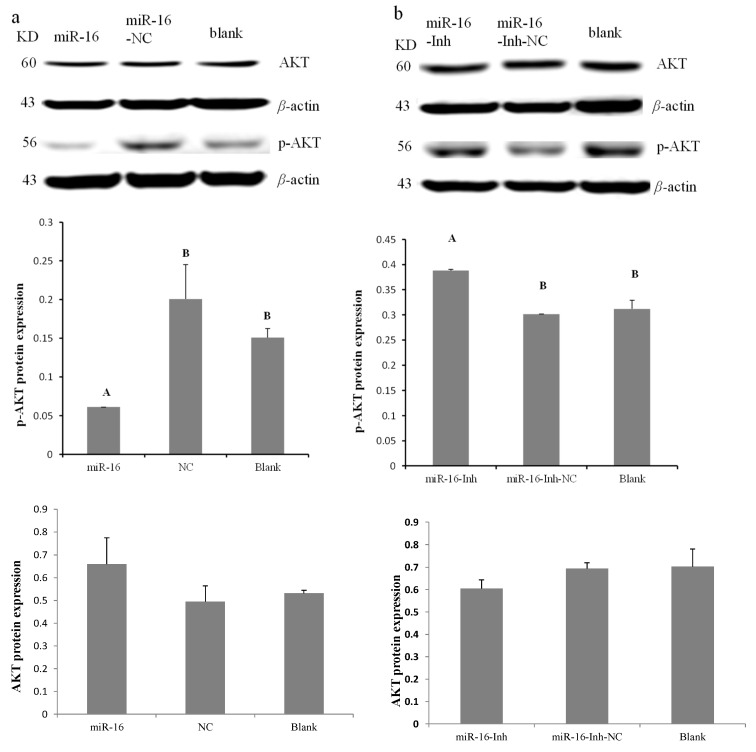
The relative protein expression of P-Akt in DF-1 cells. The protein expression of P-Akt in DF-1 cells was measured using Western blot. A mock transfection served as the blank. (**a**) Relative expression of p-Akt protein with gga-miR-16-5p overexpression. (**b**) Relative expression of p-Akt protein with gga-miR-16-5p inhibitor. All data from the triplicate experiments carried out independently were adopted as mean value ± SD. The significant difference (*p* < 0.01) is expressed as capital letters and no difference (*p* > 0.05) as the same capital letters.

To identify whether gga-miR-16-5p dysregulation alters PI3K/Akt pathway activity in DF-1 cells, gga-miR-16-5p, miR-16-Inh, miR-16-NC, and miR-16-Inh-NC were transfected into cells for 48 h. Western blotting results indicated that the level of p-Akt was effectively decreased by gga-miR-16-5p overexpression, while showing negligible effects on total Akt in DF-1 cells (Figure 6a). On the contrary, the expression of p-Akt was strongly increased by gga-miR-16-5p inhibitor but not total Akt (Figure 6b). Therefore, with these data, we could say that in DF-1 cells gga-miR-16-5p targets PIK3R1 to repress the PI3K/Akt pathway.

### 2.5. Overexpression of gga-miR-16-5p Decreases NF-κB and TNF-α Production In Vitro

**Figure 7:**
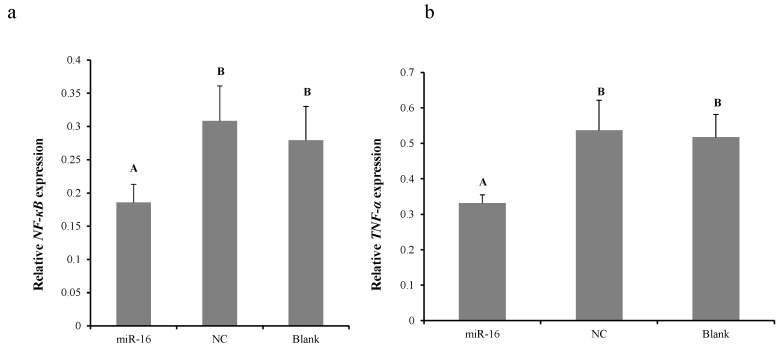
The relative expression of NF-κB (**a**) and TNF-α (**b**) mRNA after the overexpression of gga-miR-16-5p in DF-1 cells. All data from the triplicate experiments carried out independently were adopted as mean value ± SD. There was no difference between groups for the same capital letters (*p* > 0.05); there was extremely significant difference between groups with different capitals (*p* < 0.01).

To explore whether gga-miR-16-5p can inhibit the secretion of TNF-α to exert its anti-inflammatory effect via modulation of PI3K/Akt/NF-κB pathway in CRD, their expression levels were examined. DF-1 cells were transfected with gga-miR-16 mimics or miR-16-NC for 48 h. Results of qPCR showed that gga-miR-16 overexpression significantly attenuated the expression of NF-κB (Figure 7a) and TNF-α in vitro (Figure 7b). This result indicated that gga-miR-16 might act in a vital role in *MG*-infected inflammatory reaction via suppression of PI3K/Akt/NF-κB pathway to reduce TNF-α secretion.

### 2.6. Upregulated gga-miR-16-5p Represses Cell Multiplication and Cycle Progression of MG-Infected DF-1 Cells by Promoting Cell Apoptosis

**Figure 8:**
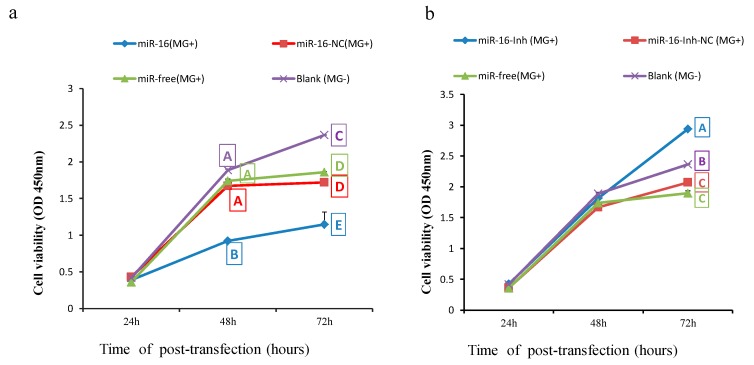
gga-miR-16-5p regulates *MG*-HS-infected DF-1 cell multiplication. (**a**) Effects of overexpressed gga-miR-16-5p on the multiplication of DF-1 cells with *MG* infected. (**b**) Effects inhibited gga-miR-16-5p on the multiplication of DF-1 cells with *MG* infected. All data from the triplicate experiments carried out independently were adopted as mean value ± SD. There are significant differences between different letters (*p* < 0.01) and no difference (*p* > 0.05) in the same capital letters.

We next explored influences of gga-miR-16-5p on multiplication, apoptosis, and cycle of cells to identify its importance. DF-1 cells were transiently transfected with gga-miR-16-5p mimics and 7 μL of *MG-HS* at a concentration of 10^10^ CCU/mL (marked as miR-16 (*MG*+)), miR-16-NC and 7 μL of *MG-HS* at a concentration of 10^10^ CCU/mL (marked as miR-16-NC (*MG*+)), 7 μL of *MG-HS* strain at a concentration of 10^10^ CCU/mL only (marked as miR-free (*MG*+)), and then uninfected DF-1 cells (marked as blank (*MG*−)), respectively. Cell Counting Kit-8 (CCK-8) assays was used to assess cell multiplication at 24 h, 48 h, and 72 h postinfection. It was found that overexpressed miR-16 (*MG*+) group significantly inhibited the cell multiplication at both 48 h and 72 h after infection in comparison with the other three groups. The cell multiplication of the blank (*MG*−) group was significantly promoted at 72 h compared with miR-16-NC (*MG*+) group and miR-free (*MG*+) group, which showed no significant difference (Figure 8a). At the same time, miR-16 inhibitor was assayed for further validation on cell proliferation. As expected, it led to a significant promotion in multiplication of cells at 72 h postinfection. We also observed a significant decreasing of the DF-1 cell multiplication when comparing miR-16-Inh-NC group (*MG*+) or the miR-free group (*MG*+) with blank control group (Figure 8b).

**Figure 9:**
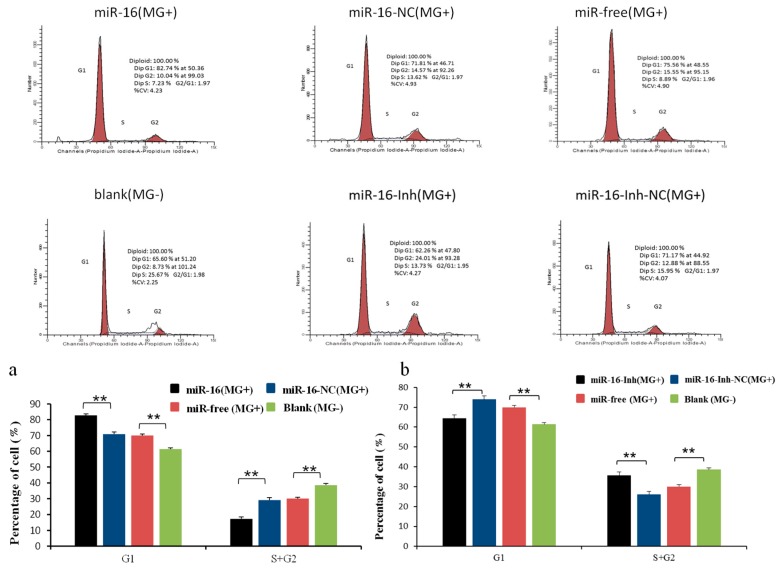
Effects of overexpression and inhibition of gga-miR-16-5p on the cell cycle of DF-1 cells with *MG* infected. (**a**) The cell cycle of DF-1 cells was remarkably suppressed by overexpression of gga-miR-16-5p. (**b**) The cell cycle of DF-1 cells was promoted by inhibition of gga-miR16-5p. All data from the triplicate experiments carried out independently were adopted as mean value ± SD. ** *p* < 0.01.

**Figure 10:**
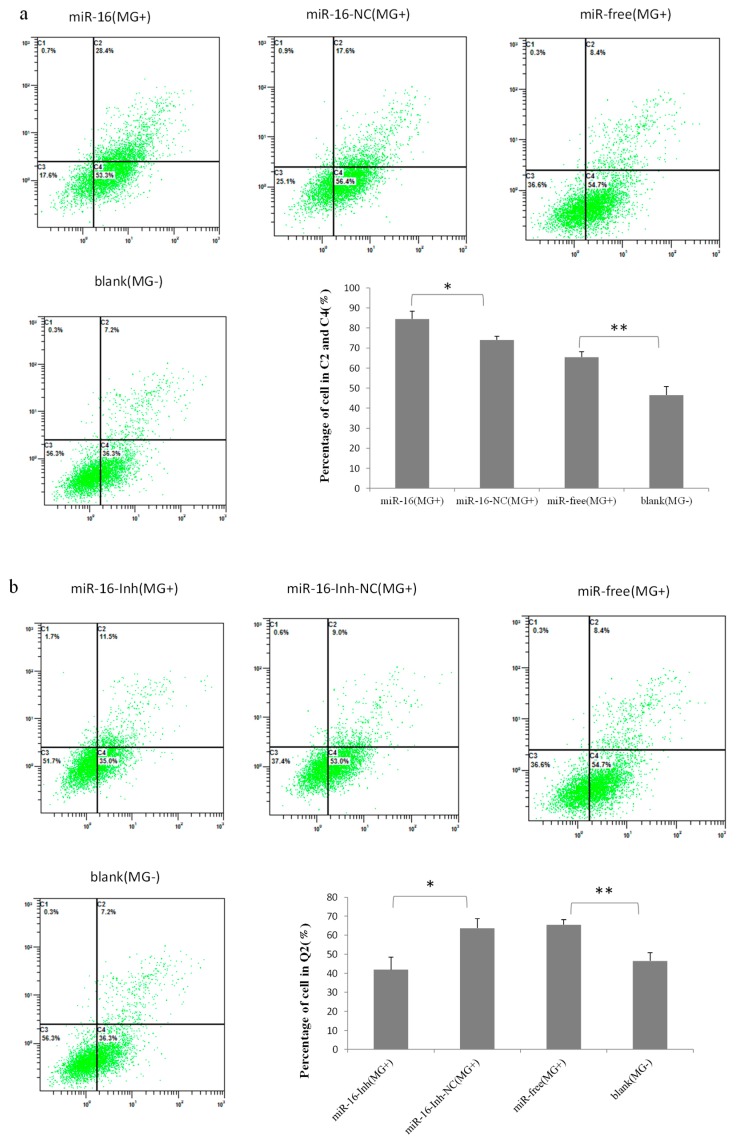
Effects of overexpression and inhibition of gga-miR-16-5p on the cell apoptosis of DF-1 cells with *MG* infected. (**a**) The cell apoptosis of DF-1 cells was remarkably promoted by overexpress of gga-miR-16-5p. (**b**) The cell apoptosis of DF-1 cells was suppressed by inhibition of gga-miR-16-5p. All data from the triplicate experiments carried out independently were adopted as mean value ± SD. ** *p* < 0.01, * *p* < 0.05.

Then, to explore how gga-miR-16-5p suppresses cell multiplication, the cell cycle, and apoptosis of DF-1, cells were assayed at 48 h postinfection by flow cytometric analysis. We found that *MG* infection disturbed mitosis by guiding the DF-1 cells arrested in the G1 cell cycle. The ectopic expression of gga-miR-16 triggered more G1 phase cell accumulation, compared with the miR-16-NC (*MG*+), or miR-free (*MG*+), or blank (*MG*−), which led to fewer cells into G2 + S phase, the blank (*MG*−) significantly raised the amount of S + G2 phases cells compared to *MG* infection groups (Figure 9a). By contrast, miR-16-Inh promoted cell cycle transition from G1 to S + G2 phase compared with the miR-16-Inh-NC (*MG*+) or miR-free (*MG*+) (Figure 9b). In the cell apoptosis analysis, overexpressed gga-miR-16 notably increased apoptosis rates compared with the miR-16-NC (*MG*+), miR-free (*MG*+), or blank (*MG*−). The blank (*MG*−) significantly reduced apoptosis compared with *MG* infection (Figure 10a). By contrast, gga-miR-16 knockdown led to apoptosis resistance compared with the miR-16-Inh-NC (*MG*+) or miR-free (*MG*+) (Figure 10b).

These findings revealed that upregulation of gga-miR-16-5p can exert anti-inflammatory effect by suppressing the PI3K/Akt/NF-κB pathway to reduce *MG*-infected cells proliferation, cycle, and enhance *MG*-infected cells apoptosis in *MG* infection.

## 3. Discussion

*MG* could cause a severe inflammatory response and an immune response in the respiratory and lung tissues of chickens [1,2]. Studies demonstrated that miRNAs might act in a pivotal role in both inflammation and avian diseases [35,36]. gga-let-7i, gga-let-7b, gga-miR-221, and gga-miR-222 might be associated with tumorigenesis induced by avian leukosis virus. [37,38]; gga-miR-130a could suppress the migration and multiplication of Marek’s lymphoma cells [39]. Our research showed the role of some miRNAs in *MG*-mediated inflammation in chicken [16,17,18,40]. At present, our study verified that gga-miR-16-5p was upregulated in both fibroblast cells and lung tissue of chicken embryos with *MG* infection, gga-miR-16-5p targets PIK3R1 to suppress *MG*-infected cells proliferation and cycle, and promotes *MG*-infected cells apoptosis by restraining PI3K/Akt/NF-κB pathway activity.

MiRNAs exert their function by binding and interfering with the target genes specifically. In our study, the upregulation of gga-miR-16-5p expression was verified in *MG*-infected lung tissue and in *MG*-infected DF-1 cells. It is confirmed that PIK3R1 was a target gene of miR-16-5p according to results of this research. PIK3R1 could regulate its downstream Akt gene, which phosphorylates target proteins through multiple downstream channels [41]. Experiments revealed that type I PI3K mainly comprised of catalytic subunit p110α as well as regulatory subunit p85α.p85α is encoded by PIK3R1 gene and acts as a critical protein [42]. PI3K/Akt is an important signal pathway of inflammation. The overexpression of miR-126 could inhibit p-Akt expression and regulate PI3K signaling by targeting PIK3R1 [43,44]. miR-16-5p was found to be involved in the regulation of the PI3K/Akt pathway in prostate patients [45]. Thus, it is rational to surmise the role of gga-miR-16-5p in *MG* infection by inhibiting PI3K/Akt signal pathway via targeting PIK3R1 gene. Our research showed that the expression of PIK3R1 and p-Akt protein in DF-1 cells was remarkably decreased following transfection with gga-miR-16-5p mimics compared with it in control groups. On the contrary, miR-16-5p inhibitor significantly increased the expression of PIK3R1 and p-Akt protein. The results were similar to the previous theoretical and experimental studies.

PI3K/AKT is involved in the regulation of cell proliferation, apoptosis and cell cycle [46]. As the center of signal transduction pathway, NF-κB is one of the important downstream molecules of AKT. In a normal state, NF-κB is combined with I-κB, and Akt can activate I-κB, causing NF-κB to be transposed into the nucleus and inducing downstream target genes related to cell multiplication, cycle progression, and apoptosis [47]. The activated PI3K/Akt pathway could activate its downstream gene NF-κB, which regulated the proliferation, migration and invasion of tumor cells [48]. The decreased expression of PI3K, p-Akt, and NF-κB can inhibit the migration of human prostate cancer cells [49]. The promotion of endothelial progenitor cell multiplication was associated with PI3K/Akt/NF-κB pathway [50].

Upregulated miR-16 was found in the colon mucosal cells of ulcerative colitis patients to activate the NF-κB pathway [51]. miR-16-5p could markedly decrease the expression of TNF-α, and miR-16-5p played an anti-inflammation role in lung inflammation caused by lipopolysaccharide [32]. Overexpression of miR-16-5p could effectively inhibit the proliferation and apoptosis of A549 lung adenocarcinoma cells [52]. Increasing the level of miR-16 could significantly inhibit the proliferation of myoblasts and promote cell apoptosis [21]. miR-16 can be regard as a tumor suppressor in human cancers, since the apoptosis, cycle, and invasion of cancerous cells may be regulated by their abnormal expression [53]. miR-16-5p may restrain multiplication and invasion of nasopharyngeal carcinoma cells via PI3K/Akt pathway [21]. Overexpression of miR-16 could effectively restrain the multiplication of DF-1 chicken embryo cells [24]. We further studied the biological function and action mechanism of gga-miR-16-5p by transient gga-miR-16-5p. The results showed elevating gga-miR-16-5p in DF-1 cells significantly inhibited NF-κB and TNF-α expression levels in our study. *MG* infection inhibited the multiplication of DF-1 cells by arresting their cell cycle at G1 phase. Upregulated gga-miR-16-5p crucially depressed the multiplication of *MG*-infected DF-1 cells by enhancing cell apoptosis and inhibiting cell cycle progression, on the contrary, the results were reversed after the gga-miR-16-5p was silenced. Our studies are consistent with previous studies.

We draw a conclusion that gga-miR-16-5p expression level was highly raised after being infected with *MG*, while expression of its target, PIK3R1, was restrained. Overexpressed gga-miR-16-5p suppressed cell multiplication and cell cycle, but promoted cell apoptosis in *MG*-infected DF-1 cells via targeting PIK3R1 to inhibit the PI3K/Akt/NF-κB pathway activation to exert anti-inflammatory effect on *MG*-infected chicken. We provided evidence for gga-miR-16-5p as a potential target to select in the treatment of CRD. We have reported the role of multiple miRNAs in MG-HS infection. Based on studies of each miRNA, we found that most of them participate in the regulation of the NF-kB pathway, the role of several important miRNAs interactions in MG-HS infection are being analyzed in depth; the maps of network regulation will be constructed to reveal the mechanism of these miRNAs in MG-HS infection.

## 4. Materials and Methods

### 4.1. Ethics Declaration

The experiments performed on chicken embryo were conducted with the approval of the Hubei Administrative Committee for Laboratory Animals (No.SYXK-2010-0029, 24 May 2010).

### 4.2. Mycoplasma Strains and DF-1 Cells

*MG-HS* was presented by the Hubei Provincial Key Laboratory of preventive veterinary medicine, Huazhong Agricultural University. *MG-HS* was cultured and its concentration was determined according to the depiction in the previous article [5]. We adopted a color-changing unit (CCU) to determinate the concentration of *MG-HS* in suspension [54]. DF-1 cells were bought from Huiying (Shanghai, China).

### 4.3. Infection Experiments

The day before transfection, cells were transplanted into 6-well cell culture plate. When the cell density was 50–60% and the cells were in good condition, the cell attack test was carried out, and the normal cell control group was set up. The original cell culture medium was sucked out and cleaned with PBS buffer for 3 times. According to the results of *MG-HS* toxicity test, the appropriate dosage was selected and the cell culture fluid (without penicillin and streptomycin) was filled to 2 mL. The 6-well culture plate was placed in the incubator at 37 °C with 5% CO_2_ to incubate for 24 h. To clean up original culture medium, the 6-well plate was washed with PBS buffer for 3 times after sucking out medium. The cells were collected by 1 mL TRIzol and stored at −20 °C.

### 4.4. The Prediction of gga-miR-16-5p Targets

To predict targets of gga-miR-16-5p what were potential, two algorithms were adopted. One is TargetScan (http://www.targetscan.org/) and the other is miRDB (http://www.mirdb.org/miRDB/). When a prediction score attains 60, it is qualified; when it attains 100, it is the best. CytoScape software was used to analyze the pathway gene association between target genes and target genes involved in different prediction software. The base pairs between gga-miR-16-5p and target sequences of PIK3R1 were further detected by RNAhybrid (http://bibiserv.techfak.uni-bielefeld.de/rnahybrid/). The AmiGO 2 software was used to analyze target genes.

### 4.5. Synthesis of RNA Oligonucleotides and Design of DNA Primers

Table S1 of Supplementary Materials includes the DNA primers that we adopted and Table S2 of Supplementary Materials shows the sequences of RNA oligonucleotides. The mimics and inhibitor of gga-miR-16-5p were from GenePharma (Shanghai, China). While the former was marked as miR-16, the latter was marked as miR-16-Inh. A mimics and inhibitor of gga-miR-16-5p were randomly selected which had not been reported to restrain any target genes of chicken to act as negative controls. While the former was marked as miR-16-NC, the latter was marked as miR-16-Inh-NC.

### 4.6. Dual-Luciferase Reporter Assay

Followed the description in double luciferase reporter gene detection kit, 5× Passive Lysis Buffer (lysate) was diluted to 1× of the working concentration with sterilized deionized water. Stop&Glo Buffer was used to dilute 50× Stop&Glo substrate (sea kidney luciferase substrate) to the working concentration of 1× (near the test), and then was foil wrapped and kept out of light. The 24-well cell culture plate was taken, the original culture solution was abandoned and the cells were cleaned two times with aseptic PBS; the cell lysate was added 100 μL in each hole, gently oscillated for 15 min, and the cells were collected into the 1.5 mL centrifuge tube. After centrifugation at 1000 r/min for 5 min, the supernatant was drawn off, and then rest at −20 °C. The 10 μL cell lysate was taken and the fluorescein substrate was added to the 50 μL fluorescein test reagent. After mixing, the luminescence value was detected in the chemiluminescence detector and the corresponding values were recorded (corresponding to the expression level of Firefly Luciferase). The centrifuge tube was taken out and the 50 μL luciferase substrate reagent was added to the test. The fixed value (corresponding to the expression level of Renilla luciferase) was standardized to calibrate the fluorescence intensity value of the sea kidney and fluorescence intensity of firefly, which was the relative expression level of the reporter gene. The experiment was triplicate in each group, and the results were averaged.

### 4.7. RNA Extraction and RT-qPCR

Trizol Reagent (Invitrogen) was adopted to extract total RNA in cells, so was it in frozen chicken embryonic lung tissues. Two-hundred microliters of chloroform was added to 1 mL Trizol cell suspension, which was subjected to severe concussion, kept still for 3 min at room temperature, and subjected to centrifugation at 4 °C at 12,000 r/min for 15 min. The supernatant was absorbed into the centrifuge tube after the treatment of the new non-RNA enzyme and isopropanol was added. Then it was mixed well by reversing the centrifuge tube several times, set statically for 15 min at 4 °C. After centrifugation at 12,000 r/min for 15 min under the condition of −20 °C the supernatant was removed. 1 mL 75% anhydrous ethanol (750 μL anhydrous ethanol + 250 μL DEPC water) was added. The precipitate substance was bounced, rinsed and then precipitated. The supernatant was abandoned after centrifugation at 4 °C at 9500 r/min. The precipitate substance was rinsed and precipitated again after 1 mL absolute ethanol was added. It was dried at room temperature for 10 min after centrifugation at 4 °C 9500 r/min. It was mixed gently and dissolved after the DEPC water was added. The total RNA concentration and purity was determined by NanoDrop-2000. RT-PCR was performed on RT-PCR machines (Bio-Rad, Hercules, CA, USA) with SYBR-green (Tiangen, Beijing, China) and a total volume of 10 μL. Relative expressions levels of gga-mir-16-5p (standardized to 5S-RNA), Akt, PI3K, and NF-κB (standardized to GAPDH) were obtained. CT (2−ΔΔCt) and IBM SPSS Statistics 20 were adopted to calculate and analyze the data. Triple experiments were repeated.

### 4.8. Western Blot

To extract total protein of DF-1 after 48 h transfection, 10 μL PMSF (100 Mm) was added into 1 mL pyrolysis solution, and 150 μL solution with PMSF was added to each hole of 6-well plate for 20–30 min. Centrifugation at 4 °C, 12,000 rpm, for 5 min. Supernatant was collected. Then 10 μg of total protein was isolated with 12% SDS polyacrylamide gel electrophoresis and transferred to polyvinylidene fluoride (PVDF) membrane, and then was blocked for 1 h in 5% skimmed milk powder solution at 25 °C. Diluted the first antibody (1:200 dilution) with sealing solution, and immersed the membrane in the first antibody dilution solution, and was kept overnight at 4 °C. Washed with TBST for three times, the membrane was immersed in TBST diluent with secondary antibody for 1–2 h. ECL reagent was adopted to evaluate protein expression level. β-actin served as control.

### 4.9. Cell Multiplication, Cycle, and Apoptosis Assays

Cell suspension was made from the logarithmic growth phase cells. 100 μL 2 × 10^4^ cells were inoculated into 96-well plates per pore, and LipofectamineTM 3000 transfection gga-miR-16-5p mimics/NC, 37 C, 5% CO_2_ cell culture box culture 24–72 h. Gga-miR-16-5p mimics or inhibitor was transfected to be overexpression or inhibition group, and 3 control groups were set up for infected cells transfected to mimics NC or inhibitor NC group, and non-miRNA group was not transfected. The normal cell group, 6 biological replicates in each group. After the first infection treatment for 2 h, cells were added each pore 10 μL CCK-8 to incubat for 1 to 4 h in the cell incubator. The light density of each well at 450 nm was read by a microplate reader (Bio-Rad, Hercules, CA, USA).

Cell suspension was made from logarithmic growth period cells, and transfected gga-miR-16-5p mimics or inhibitor as overexpression or inhibition group, and 3 control groups were set up, the infected cells were transfected to mimics NC or inhibitor NC group, and no short nucleotide fragment group and normal cell group were not transfected; each group comprised of 3 biological duplicates (6 orifice plates). When the cell density reached 50–60% and the cell condition was good, infection of 7 μL *MG-HS* (10^10^ CCU/mL) was carried out. Two hours later, the corresponding groups were transfected according to the instruction of LipofectamineTM 3000. Cells were collected 48 h later, washed with PBS once, 2000 r/min, 5 min, and the cell concentration was made to 1 × 10^6^/mL, then 1 mL of single-cell suspension was taken. The supernatant was removed after centrifugation, 500 μL 70% cold ethanol was added from 2 h to 12 h, stored at 4 °C, and washed with PBS before dying. The flow cytometry was used to detect the red fluorescence at 488 nm excitation wavelength. The cell apoptosis was analyzed similarly by annexin V, FITC apoptosis detection kit (DOJINDO, Shanghai, China).

### 4.10. Statistical Analysis

All data came from the experiments which were carried out independently at three times were mean value ± standard deviation (SD). Student’s *t*-test was adopted to analyze statistically differences. Significant differences were indicated as * *p* < 0.05, ** *p* < 0.01.

## Supplementary Materials

Supplementary materials can be found at https://www.mdpi.com/1422-0067/20/5/1036/s1.
