# Supplementary material for: Upregulated gga-miR-16-5p Inhibits the Proliferation Cycle and Promotes the Apoptosis of MG-Infected DF-1 Cells by Repressing PIK3R1-Mediated the PI3K/Akt/NF-κB Pathway to Exert Anti-Inflammatory Effect
Source: Int J Mol Sci. 2019 Feb 27;20(5):1036. doi: 10.3390/ijms20051036 (PMC6429190; doi:10.3390/ijms20051036)
Supplement: Supplementary file 1 [file ijms-20-01036-s001.pdf]

Supplementary materials

Table S1. Sequences of DNA primers

Name	Primer Sequences of DNA primers	Accessin No.
	**Primers for 3'-UTR Cloning**	
PIK3R1 3'-UTR-F	GACCTCGAGGCACTAGACCTT	XM-015277626.1
PIK3R1 3'-UTR-R	TTAGCGGCCGCTTTACCCTCCAC	XM-015277626.1
Mut-PIK3R1 3'-UTR-F	TAGCTGCCCAAGATATGGCTATTTTTTTGTTTCTATTCTTGTTTTTAAAAATAAAGC	XM-015277626.1
Mut-PIK3R1 3'-UTR-R	GAATAGAAACAAAAAAATAGCCATATCTTGGGCAGCTAATGCAGTCTCA	XM-015277626.1
	**Primers for RT-qPCR**	
RT-gga-miR-16-5p	CTCAACTGGTGTCGTGGAGTCGGCAATTCAGTTGAGCACCAATA	MIMAT0001116
gga-miR-16-5p-F	CCTGGTAGGTAGCAGCACGTAAATA	MIMAT0001116
gga-miR-16-5p-R	ACTGGTGTCGTGGAGTCGGC	MIMAT0001116
gga-5s-rRNA-F	CCATACCACCCTGGAAACGC	
gga-5s-rRNA-R	TACTAACCGAGCCCGACCCT	
PIK3R1-F	TATTTGAAGAGCAGTGCCAGAC	XM-015277626.1
PIK3R1-R	TGCTGTTCATTCGCTTGTCTAT	XM-015277626.1
AKT-F	AAAACAGAGCGACCAAAGCC	NM-205055.1
AKT-R	TGTCTGCTACAGCCTGGATTG	NM-205055.1
NF-κB-F	GCCAGGTTGCCATCGTGT	NM-205129
NF-κB-R	CGTGCGTTTGCGCTTCTC	NM-205129
GAPDH-F	GAGGGTAGTGAAGGCTGCTG	NM-204305
GAPDH-R	CACAACACGGTTGCTGTATC	NM-204305
TNF-α-F	CAGGACAGCCTATGCCAACAAG	XM-015294124
TNF-α-R	AACAACCAGCTATGCACCCCAG	XM-015294124

Table S2. Sequences of RNA oligonucleotides

Name	Sequences(5'-3')
gga-miR-16-5p mimics	UAGCAGCACGUAAAUAUUGGUG
	CCAAUAUUUACGUGCUGCUAUU
gga-miR-16-5p NC	UUCUCCGAACGUGUCACGUTT
	ACGUGACACGUUCGGAGAATT
gga-miR-16-5p inhibitor	CACCAAUAUUUACGUGCUGCUA
gga-miR-16-5p inhibitor-NC	CAGUACUUUUGUGUAGUACAA
